# Time trends in the burden of scabies from 1990 to 2021, and projections to 2050: Insights based on the Global Burden of Disease Study 2021

**DOI:** 10.1371/journal.pntd.0014237

**Published:** 2026-07-10

**Authors:** Fangqin Peng, Wenjie Hao, Yating Peng

**Affiliations:** 1 Department of Dermatology, Nanchang university Second Affiliated Hospital, Jiangxi, PR China; 2 Department of Health Toxicology, School of Public Health, Shanxi Medical University, Shanxi, PR China; Federal University of Ceará, Fortaleza, Brazil, BRAZIL

## Abstract

**Background:**

Scabies is a parasitic skin disease known since ancient times, yet it remains a persistent and significant global health challenge in the modern era affecting populations around the world. This study aims to assess the global burden of scabies across all age groups from 1990 to 2021 and predict epidemiological trends up to 2050.

**Methods:**

The study utilized data from the Global Burden of Disease Study 2021. Data from 204 countries and territories were stratified by sociodemographic index, gender, age. Age-standardized rates per 100,000 population were calculated for prevalence, incidence, and disability-adjusted life years, with 95% uncertainty intervals. Trends were assessed using Joinpoint regression models to compute the annual percent change. A time-series analysis-based prediction model was applied to estimate age-standardized incidence rates up to 2050.

**Findings:**

In 2021, the global incidence and prevalence of scabies in both sexes were 622.4 million (95% UI 556.2-695) and 206.5 million (95%UI 184.1-231.7) respectively. The age-standardized prevalence rate was 2666.47 per 100,000 population (95% UI: 2368.2–2994.5), and the age-standardized incidence rate was 8049.53 per 100,000 (95% UI: 7165.24–9024.24). The disability-adjusted life years reached 68.72 per 100,000 (95% UI: 38.3–113.2). Joinpoint regression analysis indicated a modest downward trend in age-standardized prevalence rate that did not reach statistical significance (p = 0.411), suggesting limited evidence for global improvement in scabies control. Age-stratified analysis revealed a distinct bimodal age distribution pattern in scabies incidence, the first at ages 5–14 and the second at 20–24, declining after age 25. Frontier analysis demonstrated that middle sociodemographic index regions carried the heaviest scabies burden, with the highest incidence and prevalence rates worldwide. According to the Auto-Regressive Integrated Moving Average time-series model, the global age-standardized incidence rate of scabies is predicted to decline overall by 2050, and these findings indicate that future trends are expected to vary substantially by region.

**Conclusions:**

Scabies remains a severe public health problem worldwide, and its incidence and disease burden demonstrate significant regional and age-related variations. The age-standardized prevalence rate in most sociodemographic index regions showed a downward trend, reflecting that the incidence of scabies has been controlled to some extent, but the prevalence rate is still very high. However, the future epidemiological trend of scabies has regional heterogeneity, public health interventions should focus on countries and territories in the high-middle sociodemographic index regions and middle sociodemographic index regions, optimizing healthcare resource allocation, improving basic health infrastructure, enhancing health education.

## Author summary

Scabies is a contagious dermatosis caused by *Sarcoptes scabiei* parasitizing in the human skin epidermis. It remains a significant global public health challenge despite being recognized as a neglected tropical disease by WHO in 2017. The disease predominantly affects tropical and resource-limited regions, with overcrowding, poverty, and inadequate sanitation serving as key risk factors. Characterized by intense nocturnal itching and skin lesions, scabies can lead to secondary bacterial infections and serious complications including impetigo and rheumatic heart disease. Using data from the Global Burden of Disease Study 2021, this study examined global scabies burden across 204 countries and territories from 1990 to 2021, with projections to 2050. The global scabies incidence reached 622.4 million cases in 2021. The burden remained highest in middle-SDI regions, which accounted for over 40% of global cases. Notably, high-middle SDI regions showed an increasing trend, while low-SDI regions declined. Frontier analysis identified substantial efficiency gaps in Pacific Island nations, whereas high-SDI countries approached optimal control. We predict a temporary rebound in 2027, offering policymakers a critical window for intervention. These findings highlight persistent regional disparities and underscore the need for targeted public health interventions and strengthened surveillance systems, particularly in middle-SDI regions facing increasing burden.

## Introduction

Scabies, caused by the parasitic mite *Sarcoptes scabiei*, was officially recognized as a neglected tropical disease by the World Health Organization (WHO) in 2017 [1]. Scabies remains a pressing global public health concern [2], affecting populations universally. The 2021 Global Burden of Disease (GBD) Study estimated the global point prevalence of scabies to be around 206.6 million, with 622.5 million annual incident cases in 204 countries. Further GBD analyses estimated that scabies caused approximately 5.3 million disability-adjusted life-years (DALYs) [3]. High-risk populations primarily reside in resource-poor low-income and middle-income countries, particularly among indigenous communities in the Pacific Islands, Central America, and northern Australia [4,5]. In these regions, community prevalence rates can reach 20–30%, with childhood prevalence as high as 40–50% [6]. Scabies usually manifests as intense itching at night with skin lesions. The signs consistent with scabies are the presence of burrows, erythematous papules, nodules, and vesicles, alongside pruritus or evidence of excoriation. Severe cases may develop secondary bacterial infections such as impetigo, potentially leading to invasive soft tissue infections, sepsis, post-streptococcal glomerulonephritis, and rheumatic heart disease [7]. To address this public health challenge, a series of mass drug administration controlled trials with ivermectin were conducted in high-prevalence settings [8–10]. Studies indicate this approach can reduce scabies prevalence by over 90% and impetigo by approximately 67% in highly endemic areas such as Fiji and the Solomon Islands [11]. Given the constraints of large-scale global studies and significant inconsistencies in prevalence measurement methods on scabies, existing researches failed to capture long-term epidemiological trends [12,13]. Compared to Li et al.’s study [3], our research identified critical turning points through Joinpoint analysis and quantified trends at each stage, which can be used to evaluate the effectiveness of public health interventions and policies. Frontier analysis revealed the gap between the actual and theoretically optimal levels of scabies control across countries under existing economic conditions. Additionally, the Auto-regressive Integrated Moving Average (ARIMA) time-series model predicted a temporary rebound in global scabies incidence in 2027, providing a more explicit timeline for precise prevention and control. The results of the study will provide policy makers with a timetable for optimizing of allocation of global health resources. In addition, the study will facilitate the formulation of active interventions to reduce the burden of scabies. These findings provide valuable epidemiological information for research purposes on prevention and treatment of scabies, and help clinicians, epidemiologists and health policy makers to optimize the allocation of medical resources and formulate more effective public health strategies.

## Methods

### Ethics statement

This secondary analysis of de-identified aggregate data qualified as non-human subjects research and was granted an institutional review board exemption from Nanchang University Second Affiliated Hospital.

### Data sources

This observational study utilized comprehensive epidemiological data from the Global Burden of Diseases, Injuries, and Risk Factors Study (GBD) 2021, encompassing 204 countries and territories [14]. Epidemiological estimates (including prevalence, incidence, disability-adjusted life year rate) were directly extracted from the GBD 2021 database. All other metrics, including Joinpoint-identified trend inflection points, ARIMA model data from 2022 to 2050, and frontier-based efficiency gaps, were calculated by the authors using the extracted data. The study adhered to the Strengthening the Reporting of Observational Studies in Epidemiology (STROBE) guidelines.

### Case definition and metrics

This study identified scabies cases through the application of International Classification of Diseases (ICD)-9 code 133 and ICD-10 code B86 [15]. We analyzed age-standardized incidence rates, prevalence, and disability-adjusted life years (DALYs) across all demographic groups. Scabies was categorized within the GBD 2021 cause group of skin and subcutaneous conditions and assigned a disability weight for disfigurement level 1 with itch/pain (0.027, 95% CI 0.015–0.042). The lay description for this level characterizes the condition as follows: “The individual has a slight, visible physical deformity that is sometimes sore or itchy, which is noticeable to others and causes some worry and discomfort.” DALYs, calculated as the sum of Years of Life Lost (YLLs) and Years Lived with Disability (YLDs), served as the primary burden metric. Given that scabies-related mortality is negligible, YLLs were assumed to be zero, rendering DALYs approximately equal to YLDs. The Sociodemographic Index (SDI), a composite measure of income per capita, educational attainment, and fertility rate, was used to assess development status. Based on SDI scores (ranging from 0 to 1), countries and regions worldwide are categorized into five tiers: high SDI (≥0.80), high-middle SDI (0.70–0.80), middle SDI (0.60–0.70), low-middle SDI (0.45–0.60), and low SDI (<0.45). In the field of public health, efficiency gaps specifically refer to disparities in the input of health resources versus health outcome benefits across different regions, healthcare systems, or populations. These gaps reflect inequalities in the effectiveness of resource allocation or interventions. This concept is commonly used to assess the equity of healthcare services and to optimize health policies.

### Statistical analysis

Data were presented as numerical counts and age-standardized rates (ASRs) per 100,000 population, with 95% uncertainty intervals (UIs). Frontier analysis was conducted using a nonparametric bootstrap-cum-minimum approach. Age-standardized DALY rates were extracted from the GBD 2021 database, merged with SDI values, and sorted by SDI ascending and DALY rate descending. To ensure robust estimation, 100 bootstrap iterations were performed by random sampling with replacement. Within each bootstrap sample, the frontier was constructed by calculating the cumulative minimum of DALY rates across the SDI spectrum, representing the lowest observed burden at each level of sociodemographic development. Super-efficient outliers were excluded using a leave-one-out cross-validation procedure. The final frontier values were obtained by averaging across all bootstrap iterations. We utilized frontier analysis to evaluate the relationship between scabies burden and sociodemographic development, to identify potential improvement margins across nations using SDI-stratified benchmarks, and to establish evidence-based minimum achievable age-standardized DALY rates.

This study employed an Auto-Regressive Integrated Moving Average (ARIMA) model to forecast trends in annual occurrence rates from 1990–2021 up to 2050. The selection of the ARIMA model was based on the following considerations. First, the GBD data constitute a continuous and complete annual time series, meeting the fundamental requirements of the ARIMA modeling. Second, compared with simple linear regression, the ARIMA model, by integrating the three components of autoregression (AR), differencing (I), and moving average (MA), can effectively capture complex trend inflections and periodic fluctuations within the data, which aligns well with the multi-phase change characteristics identified by Joinpoint analysis. Third, this model has been extensively validated for disease burden forecasting in the public health, and its reliability is well-established [16]. Model fitting was performed using the `auto.arima` function in R software (version 4.3.2; R Foundation for Statistical Computing) to automatically select the optimal parameter combination. This algorithm is based on the principle of minimizing the Akaike Information Criterion (AIC) or the Bayesian Information Criterion (BIC), thereby balancing goodness-of-fit with model parsimony and avoiding over-fitting. Model diagnostics included Ljung-Box test (lag = 10) using the function to assess whether residuals exhibited white noise (p > 0.05 indicates no significant auto-correlation) [17]. To account for forecast uncertainty, 95% confidence intervals (95% CI) were automatically generated by the forecast function based on model residual variance and the forecast horizon.

## Result

### Trends at global, regional and national levels

**Fig 1:**
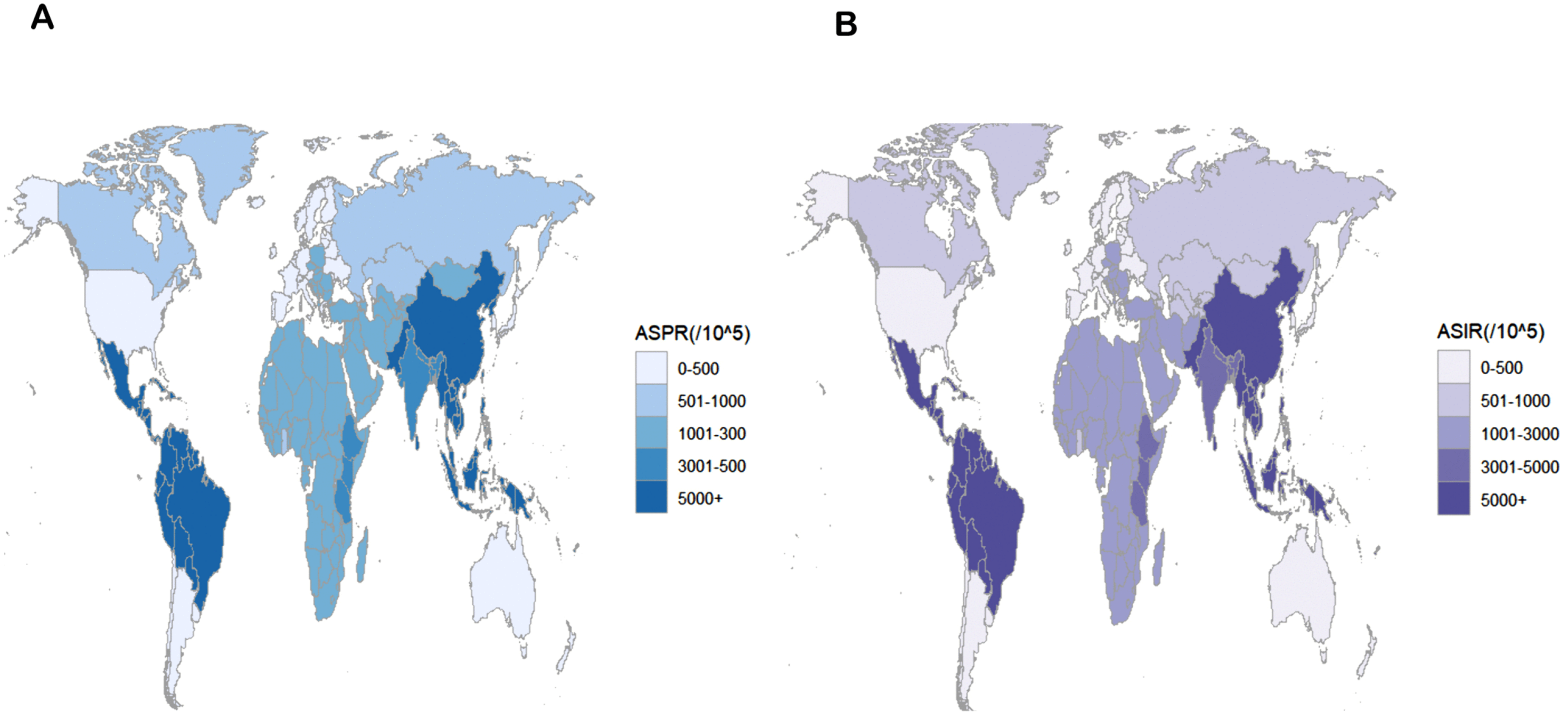
The global distribution of age standardized prevalence (A) and incidence (B) rate of scabies for both sexes. Abbreviations: ASPR:age-standardized prevalence rate; ASIR: age-standardized incidence rate https://CRAN.R-project.org/package=rnaturalearth. Basemap data source: Natural Earth, http://www.naturalearthdata.com, public domain.

The global burden of scabies, including the prevalence and incidence patterns, remains substantial (Table 1 and Fig 1). Furthermore, in 2021, an estimated 622.5 million (95% UI: 556.23–695 million) new scabies cases occurred worldwide. Specifically, the age-standardized incidence rate (ASIR) was 8,049.5 per 100,000 population (95% UI: 7165.2–9024.2). Additionally, the prevalent cases reached 206.6 million (95% UI: 184.2–232.2), which corresponds to an age-standardized prevalence rate (ASPR) of 2,666.47 per 100,000 population (95% UI: 2368.2–2994.5). Notably, the highest prevalence and incidence rates were typically observed in tropical regions and middle-SDI countries, such as Fiji (ASPR; 8,769.3 per 100,000 population: 95% UI: 7,754.1–9,833.87).

**Table 1:** The global burden of scabies by SDI groups in 2021.

Group by SDI	Incidence		Prevalence		DALYs (Disability-Adjusted Life Years)	
	No.(95%UI)	Age-specific rate per 100000(95%)	No.(95%UI)	Age-specific rate per 100000(95%)	No.(95%UI)	Age-specific rate per 100000(95%)
**Global**	**622473574 (556234883,694992043)**	**8049.53 (7165.24,9024.24)**	**206549645 (184175477,231740498)**	**2666.47 (2368.24,2994.51)**	**5315519 (2968359,8740280)**	**68.72 (38.33,113.25)**
**High SDI**	**17877244 (16076500,19879409)**	**1737.04 (1552.82,1937.97)**	**5938591 (5327897,6604561)**	**575.51 (513.47,647.22)**	**151885 (86118,243655)**	**14.87 (8.37,24.17)**
**High-middle SDI**	**101065671 (90201546,112618992)**	**8312.81 (7413.35,9310.96)**	**33557796 (29927726,37366633)**	**2748.91 (2443.64,3093.74)**	**861980 (486463,1399734)**	**71.14 (39.84,117.17)**
**Middle SDI**	**268680297 (240226207,299946245)**	**11409.13 (10174.5,12796.17)**	**89185732 (79610620,99815563)**	**3774.71 (3361.32,4223.75)**	**2294380 (1287907,3754027)**	**97.32 (54.38,159.96)**
**Low-middle SDI**	**166777845 (146956624,189229173)**	**8521.66 (7528.21,9581.87)**	**55282581 (48210375,63002854)**	**2820.52 (2488.56,3176.97)**	**1424193 (790972,2354784)**	**72.4 (40.25,119.19)**
**Low SDI**	**67501060 (58688542,77417424)**	**5648.39 (5034.53,6348.26)**	**22395192 (19434928,25751101)**	**1879.69 (1662.82,2122.73)**	**578195 (319569,959506)**	**48.11 (26.8,78.98)**

Abbreviations: DALYs, Disability-Adjusted Life Years; UI, uncertainty interval; SDI, social demographic index.

**Fig 2:**
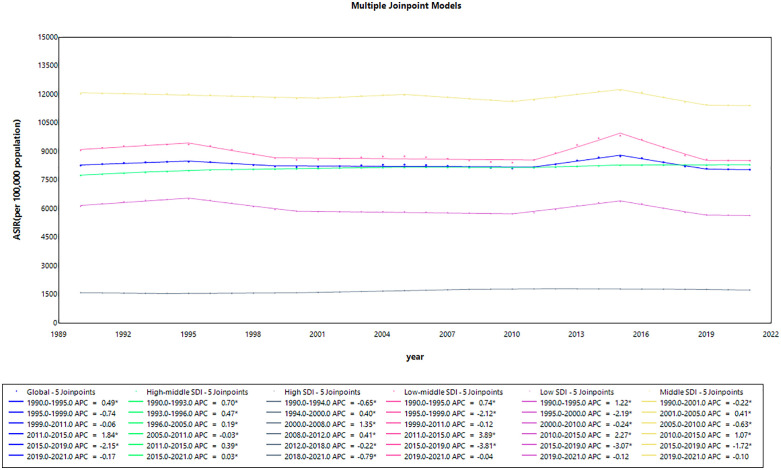
Age-standardized incidence rate per 100 000 for scabies in global and different SDI regions from 1990 and 2021. Global: blue, High SDI: gray, High-middle SDI: green, Low-middle SDI: pink, Middle SDI: yellow, Low SDI: rose.

We used the Joinpoint regression model to analyze the trend of scabies ASIR globally and across different SDI regions, finding differences in trends among SDI regions. Globally, the scabies ASIR burden demonstrated a modest downtrend (AAPC = -0.0966; 95% CI: -0.3267 to 0.134; p = 0.411) (Figs 2 and S1A), which was not statistically significant. This lack of significant decline may reflect persistent surveillance gaps, variable diagnostic practices across regions, and under-reporting in resource-limited settings, rather than genuine stabilization of the disease burden.

The trend was characterized by a slight increase from 1990 to 1995 (APC = 0.49), followed by a gradual decline from 1995 to 1999 (APC = -0.74) and from 1999 to 2011 (APC = -0.06). A subsequent rebound occurred between 2011 and 2015 (APC = 1.84), before another downward movement from 2015 to 2019 (APC = -2.15) and a mild decrease from 2019 to 2021 (APC = -0.17).

High-SDI regions also showed a significant increasing trend (AAPC = 0.2720; 95% CI: 0.2323 to 0.3116; p < 0.001) (Figs 2 and S1B). An initial gradual decrease was observed from 1990 to 1994 (APC = -0.65), followed by a steady climb from 1994 to 2000 (APC = 0.40), a more marked increase from 2000 to 2008 (APC = 1.35), and a moderate rise from 2008 to 2012 (APC = 0.41). The trend then reversed, showing a slight decline from 2012 to 2018 (APC = -0.22) and a more pronounced drop from 2018 to 2021 (APC = -0.79).

High-middle-SDI regions exhibited the most notable upward trend (AAPC = 0.2186; 95% CI: 0.2026 to 0.2347; p < 0.001) (Figs 2 and S1C). The period 1990–1993 saw a moderate increase (APC = 0.70), followed by a continued yet slower rise from 1993 to 1996 (APC = 0.47) and 1996–2005 (APC = 0.19). A slight decline occurred between 2005 and 2011 (APC = -0.03), after which the trend reversed upward from 2011 to 2015 (APC = 0.39) and remained nearly stable from 2015 to 2021 (APC = 0.03).

Middle-SDI regions exhibited a statistically significant decreasing trend (AAPC = -0.1861; 95% CI: -0.2774 to -0.0946; p < 0.001) (Figs 2 and S1D). A gradual decline was observed from 1990 to 2001 (APC = -0.22), followed by a moderate rise from 2001 to 2005 (APC = 0.41). The trend then reversed with a moderate decline from 2005 to 2010 (APC = -0.63), and again shifted upward from 2010 to 2015 (APC = 1.07). A marked decrease followed from 2015 to 2019 (APC = -1.72), ending with a minor downturn from 2019 to 2021 (APC = -0.10).

Low-middle-SDI regions displayed the most considerable declining trend (AAPC = -0.2129; 95% CI: -0.5719 to 0.1474; p = 0.2464) (Figs 2 and S1E), though it was not statistically significant. A moderate upward movement occurred between 1990 and 1995 (APC = 0.74), followed by a sharp decrease from 1995 to 1999 (APC = -2.12) and a mild decline from 1999 to 2011 (APC = -0.12). A strong rebound was observed from 2011 to 2015 (APC = 3.89), succeeded by a rapid drop from 2015 to 2019 (APC = -3.81) and a minimal decrease from 2019 to 2021 (APC = -0.04).

Low-SDI regions showed a significantly pronounced decline (AAPC = -0.2858; 95% CI: -0.4061 to -0.1654; p < 0.001) (Figs 2 and S1F). An initial moderate increase occurred from 1990 to 1995 (APC = 1.22), followed by a sharp downturn from 1995 to 2000 (APC = -2.19) and a gentle decline from 2000 to 2010 (APC = -0.24). A subsequent rebound took place between 2010 and 2015 (APC = 2.27), before another sharp decrease from 2015 to 2019 (APC = -3.07) and a slight reduction from 2019 to 2021 (APC = -0.12).

### Group by SDI region, gender and age

Our study reveals a strong correlation between disease burden distribution and SDI levels (Table 1), with middle-SDI regions bearing the heaviest burden. It is evident that these regions demonstrate the world’s highest incidence rate (11,409.13 per 100,000; 95% CI 10,174.5 to 12,796.2), prevalence rate (3,774.71 per 100,000; 95% CI 3,361.3 to 4,223.8), and DALY rate (97.32 per 100,000; 95% CI 54.4 to 160). Collectively, these figures account for over 40% of global totals in both incident cases (269 million; 95% CI 240.2 to 300) and prevalent cases (89.2 million; 95% CI 79.6 to 99.8). Following closely, high-middle SDI regions rank second only to middle-SDI in scabies burden, with the incidence rate (8,312.81 per 100,000; 95% CI 7,413.35 to 9,310.96), prevalence rate (2,748.91 per 100,000; 95% CI 2,443.64 to 3,093.74), and DALY rate (71.14 per 100,000; 95% CI 39.84 to 117.17) significantly surpass global averages. While the burden of scabies disease is below the global average in both low-middle and low-SDI regions, it nevertheless accounts for a substantial number of cases, with 167 million new cases in low-middle SDI regions and 67.5 million in low-SDI regions.

An analysis of the scabies disease burden revealed persistent gender differences, with males demonstrating not only greater absolute case numbers but also significantly higher age-standardized rates of incidence, prevalence, and DALYs than their female counterparts. This trend was evident across all global and SDI-stratified populations.

**Fig 3:**
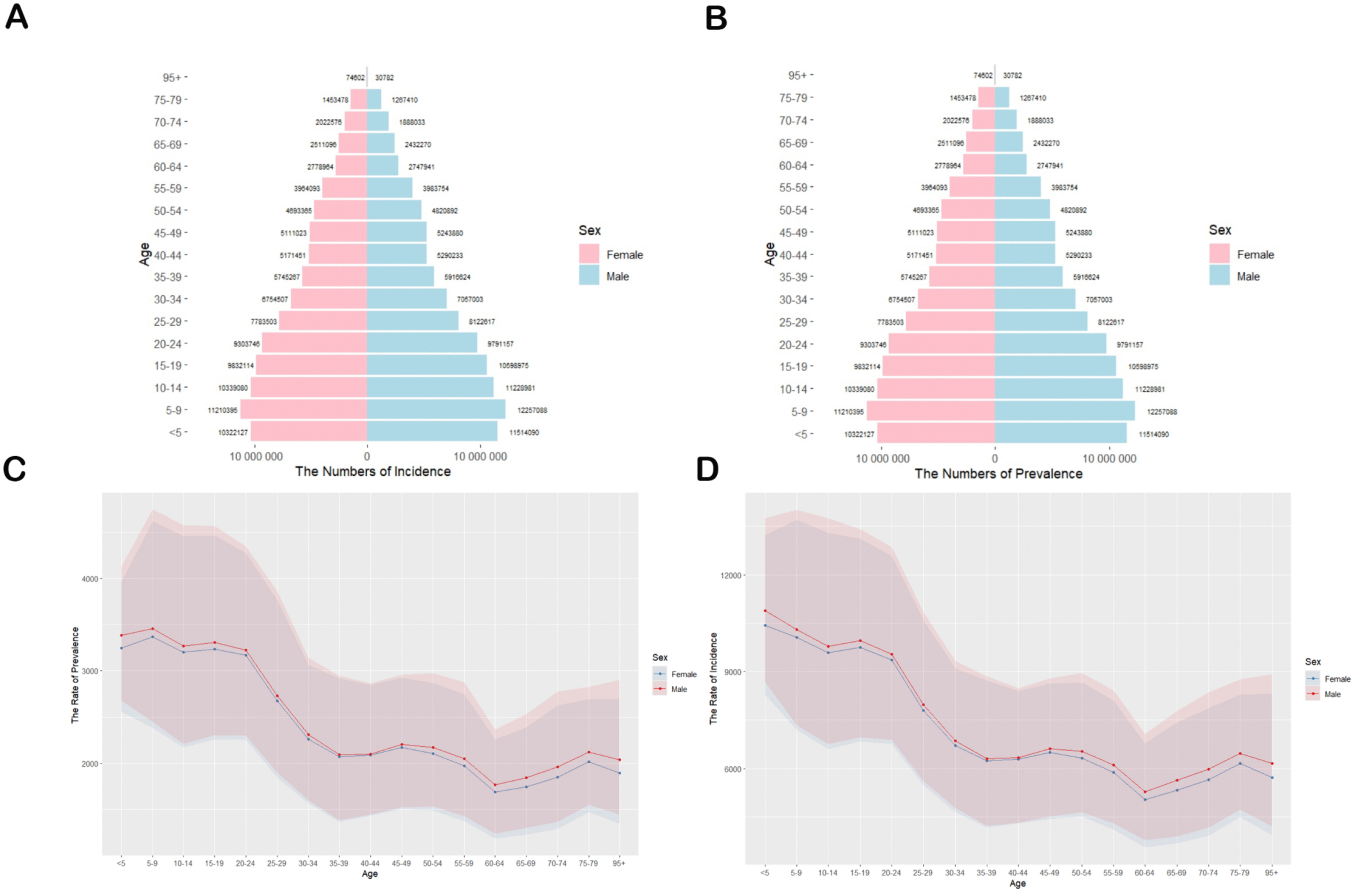
Age and sex distribution of global incidence and prevalence of male and scabies in 2021. The numbers of scabies incidence **(A)** and prevalence **(B)** for both sexes in 2021. The age specific of scabies prevalence **(C)** and incidence **(D)** for both sexes in 2021. (Red bar and line represent female, blue bar and line represent male.).

A through epidemiological analysis has been conducted, which has revealed that the incidence of scabies follows a pronounced bimodal age distribution pattern, characterized by two distinct peaks. The first peak occurs predominantly among school-aged children(5–14 years), while the second emerges among 20–24 age group (Fig 3A and 3B). Notably, elderly populations exhibit relatively stable incidence rates, although certain datasets suggest a marginal but potentially significant upward trend in these age groups (Fig 3A–3D).

### Frontier analysis

**Fig 4:**
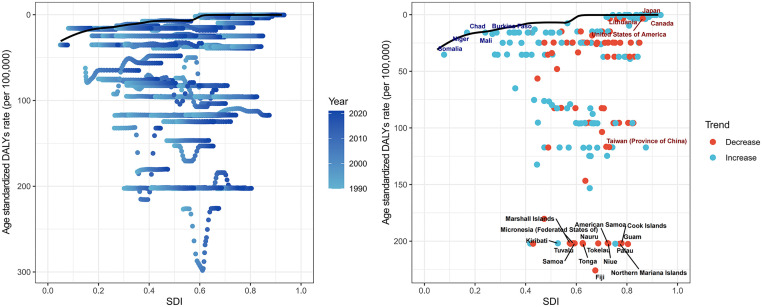
Frontier analysis of age-standardized Disability-Adjusted Life Year (DALY) rates for scabies by Socio-demographic Index (SDI) in 2021. The x-axis represents the Socio-demographic Index (SDI), a composite measure of social development ranging from 0 to 1. The y-axis displays the age-standardized DALY rate per 100,000 population. The solid line denotes the efficient frontier, representing the lowest achievable DALY rate at each SDI level. The color gradient in Panel A illustrates the progression of years, from the lightest shade representing 1990 to the darkest shade representing 2021. In Panel B, each circle represents an individual country or territory. Red circles indicate countries with a decreasing trend in DALY rates during the study period; blue circles indicate countries with an increasing trend. The vertical distance between a circle and the frontier line represents the efficiency gap, reflecting the potential for reducing the disease burden given the country’s current socioeconomic conditions.

We employed frontier analysis to quantify the attainable efficiency gains in scabies control. The analysis drew on data from 1990 to 2021, and utilized the metrics of ASDR and SDI to inform the exploration of optional efficiency. High-SDI regions (SDI > 0.8), including Canada, the United States, and Japan, exhibited minimal efficiency gaps (eff_diff<5) (Fig 4), where their actual DALY rates (val) nearly coincided with the theoretical optimal levels (frontier), signifying that these nations have attained a state of optimal efficiency in scabies control, considering their prevailing socioeconomic circumstances. Pacific island nations exhibited significant efficiency gaps. Of the fifteen regions exhibiting the most significant efficiency gaps, those belonging to the Pacific Island demonstrated the most pronounced trends. Notably, Fiji ranked first with an efficiency gap of 225.51 DALYs/100,000. Despite exhibiting middle-range SDI levels (0.53-0.80), the observed DALY rates (201–226) significantly exceeded the theoretical frontier values (0.3-6.9), thereby highlighting substantial deficiencies in health system performance. The efficiency exhibited by Low-SDI countries was found to be remarkably high. Nations such as Somalia (SDI = 0.08) and Niger (SDI = 0.17) displayed the minimal efficiency gaps (eff_diff<0.2), with their actual DALY rates (15.8-35.3) closely approaching theoretical frontiers. This may reflect that in these regions with extremely low development levels, the scabies burden has already reached the baseline unavoidable level.

### The trends in the burden of scabies up to 2050 by ARIMA forecast

**Fig 5:**
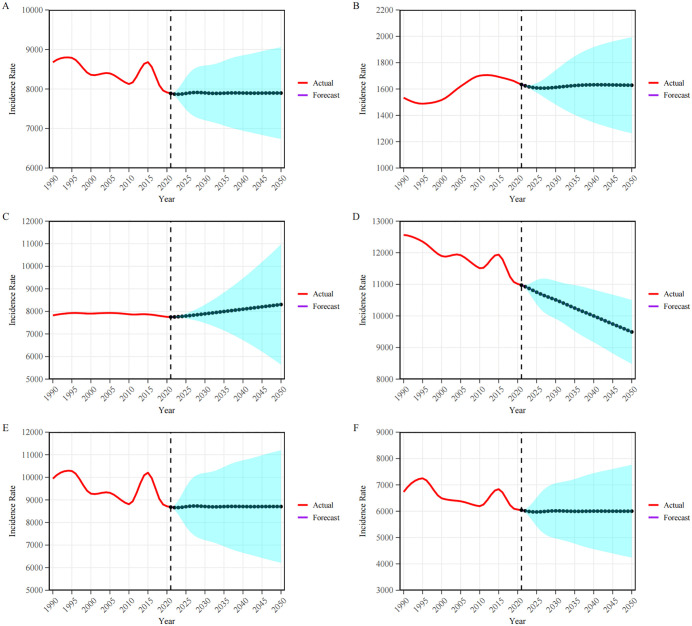
Time trends of ASIR in scabies in global and different SDI regions from 1990 to 2050. Solid lines represent observed historical data (1990–2021) from the GBD 2021 study; dashed lines and shaded regions represent model-projected trends (2022–2050) with 95% CI. The Y-axis indicates ASIR per 100,000 population; the X-axis represents calendar year. (A) Global, (B) High SDI, (C) High middle SDI, (D) Middle SDI, (E) Low middle SDI, (F) Low SDI.

The predictive analysis conducted using the ARIMA time series model suggests that the ASIR of scabies, which reached its peak in 2015 at 8752.67 per 100,000, has been on a general decline since then. Interestingly, the model forecasts a potential temporary resurgence in the global scabies ASIR in 2027, with an estimated rate of 8460.05 per 100,000 (95% CI 8177.13 to 8742.98) (Fig 5A), forming a secondary peak. A stratified analysis by the SDI uncovers significant regional variations (P < 0.05), with middle-high SDI regions displaying a continuous upward trend, projected to hit 8937.91 per 100,000 by 2050 (95% CI 6760.29–11115.52) (Fig 5C). Middle SDI regions, although decreasing slowly from their 2015 peak of 12213.38 per 100,000, still maintain the highest global incidence rates (Fig 5D). Middle-low SDI regions also exhibit a gradual decline but are expected to stay above 8900 per 100,000 in the long term (Fig 5E). In contrast, while the ASIR of high SDI regions remains steady (Fig 5B), it is significant that low SDI regions are the only ones showing a consistent and marked downward trend (Fig 5F).

## Discussion

With the rapid growth of the global population, our results show an increase in the absolute number of scabies cases, while the global incidence rate demonstrates a declining trend (Fig 2). The geographical distribution of scabies burden is closely correlated with SDI levels, with middle-SDI regions bearing the heaviest burden, followed by middle-high SDI regions, while high-SDI regions show the lowest burden. Frontier analysis confirms this pattern, demonstrating that high-SDI regions have achieved optimal scabies control efficiency under their current socioeconomic conditions.

Notably, we observed that in some high-SDI countries like the United States and Australia, scabies prevalence and incidence rates exceed those of other high-SDI nations and even many resource-limited tropical countries (Fig 1 and Table 1). This phenomenon may be related to the sporadic nature of cases in affluent regions, where public health efforts primarily focus on controlling outbreaks within institutional settings, particularly healthcare facilities and long-term care residences for the elderly [6]. However, this explanation remains speculative and requires further investigation. In contrast, low- and middle-income countries face substantially greater disease burdens, where limited access to effective treatments and crowded living conditions facilitate transmission.The highest prevalence rates are reported in tropical and subtropical zones, particularly in Pacific Island communities and Central American regions [4,5]. A prominent example is the Ethiopian epidemic, ongoing since 2015, which has affected an estimated one million people [18]. High transmission rates are typically associated with overcrowding, with outbreaks commonly occurring in schools, prisons, and refugee camps [19]. Changes in environmental or sociopolitical conditions may contribute to scabies epidemics [20]. A 10-year retrospective study conducted in Bologna, Italy revealed a significant increasing trend in scabies incidence with distinct seasonal peaks in recent years. The study suggested that environmental and social factors, particularly increased tourist mobility and substandard living conditions, may serve as potential risk factors for scabies transmission [21].

Mass drug administration trials with ivermectin in island communities have demonstrated the potential for population-level control in high-prevalence areas [10]. While mass treatment shows clear advantages in such settings, intensified case management strategies may be more appropriate in low-prevalence environments. Our study reveals the unexpected finding that low-SDI regions exhibit scabies burdens below global averages (Table 1). Frontier analysis suggests this may reflect these regions reaching a “baseline unavoidable level” due to extremely low development. In these under-performing health systems, accurately diagnosing scabies remains challenging for doctors, which results in under-diagnosis and under-reporting. The consensus criteria of scabies may promote diagnostic standardization and improve epidemiological data reliability [7].

Contrary to existing findings suggesting no gender disparity [13,15], we observed significant sex differences in scabies burden, with males showing higher rates than females. It seems to have early signs in some areas [22,23]. The incidence displays a distinct bimodal age distribution, peaking first in adolescents age 5 to age 14 and again in young adults age 20 to age 24. This aligns with global research showing children and young adults bear the greatest burden, likely reflecting scabies’ transmission through close contact. Children in schools and young adults in dormitories, military barracks, or early family living situations face elevated exposure risks, with sexual contact potentially contributing to transmission.

Currently, scabicides (e.g., ivermectin, permethrin) remain the only approved treatments. Ivermectin, the sole oral option, is primarily used for crusted scabies but has limitations: its short cutaneous half-life often requires multiple doses, and it’s not approved for children under 15 kg [2,24,25]. These treatment constraints particularly affect infants and children who are vulnerable with developing immunity that cannot acquire lasting protection after infection. The toxicity concerns of scabies insecticide may contribute to the high incidence in this age group. Various vaccine development efforts (whole-mite extracts, DNA vaccines [26], recombinant fusion proteins [27]) are underway, which may revolutionize global scabies control strategies in the future [28]. Frontier analysis highlights that Pacific Island nations should prioritize improving primary healthcare accessibility and implementing mass drug interventions (such as ivermectin-based population treatment). The experience of high-SDI countries demonstrates that enhancing health insurance coverage and diagnostic standardization can eliminate avoidable scabies burden. The apparently efficient performance of low-SDI countries may mask underlying surveillance deficiencies, necessitating strengthened disease monitoring systems.

We predicted the trends in the global burden of scabies and across different SDI regions over the next 25 years. Based on the ARIMA model, it is projected that by 2050, the global burden of scabies may experience a minor peak. The ASIR of scabies in middle SDI regions is declining slowly but remains the highest globally. The middle-high SDI regions show a continuous upward trend, while the middle-low SDI regions exhibit a similar pattern of gradual decline. High SDI regions remain stable, and low SDI regions are the only areas demonstrating a consistent and significant downward trend. These results indicate that future scabies prevention and control efforts should prioritize middle-high, middle, and middle-low SDI regions, as these areas continue to face a significant disease burden. It is recommended to develop differentiated prevention and control strategies tailored to the specific characteristics of each SDI region.

### Limitations of the study

This study based on the GBD 2021 database that provides a comprehensive analysis of the global burden of scabies. However, several limitations should be acknowledged. Firstly, under-reporting and diagnostic limitations in low-SDI regions are clearly major limitations. A study suggested that the “ScAbIeS” tool could be used to diagnose scabies at the community level in resource-limited settings by Community Healthcare Workers with appropriate training [29]. Secondly, the precision of the estimates may be contingent upon the quality and accessibility of data sources across different geographical locations. High-income countries such as Japan and Germany lack direct surveillance systems and rely on health insurance records, substantially underestimating the true prevalence. Thirdly, the GBD has limited ability to capture clinical sub-types, risk factors, and complications of scabies. Furthermore, the disability weight assigned to scabies only reflects the direct effects of skin infection, not secondary disability [15]. These limitations underscore the urgent need to improve global scabies surveillance systems. Future efforts should focus on establishing more comprehensive data collection methods, particularly strengthening case reporting in primary healthcare settings, and developing assessment frameworks that better reflect the multidimensional impact of scabies.

In conclusion, the global burden of scabies remains significant, with middle SDI regions bearing the heaviest burden. While overall trends show a decline in incidence and prevalence by 2050, disparities persist: middle-high SDI regions would face rising rates, whereas low SDI regions would exhibit marked improvements. Gender and age disparities highlight vulnerable groups, emphasizing the need for targeted interventions. Strengthening surveillance and tailored control strategies are crucial to address regional variations and achieve global scabies reduction.

## Supporting information

S1 Fig Age-standardized incidence rate per 100 000 for scabies in global and different SDI regions from 1990 and 2021. (TIF)

S1 Table The global burden of scabies by SDI groups in 2021. (DOCX)

S1 Appendix Table for Fig 1A. The global distribution of age standardized prevalence rate of scabies.
(XLSX)

S2 Appendix Table for Fig 1B. The global distribution of age standardized incidence rate of scabies.
(XLSX)
