# Supplementary figures and images for: Time trends in the burden of scabies from 1990 to 2021, and projections to 2050: Insights based on the Global Burden of Disease Study 2021
Source: PLoS Negl Trop Dis. 2026 Jul 10;20(7):e0014237. doi: 10.1371/journal.pntd.0014237 (PMC13412055; doi:10.1371/journal.pntd.0014237)

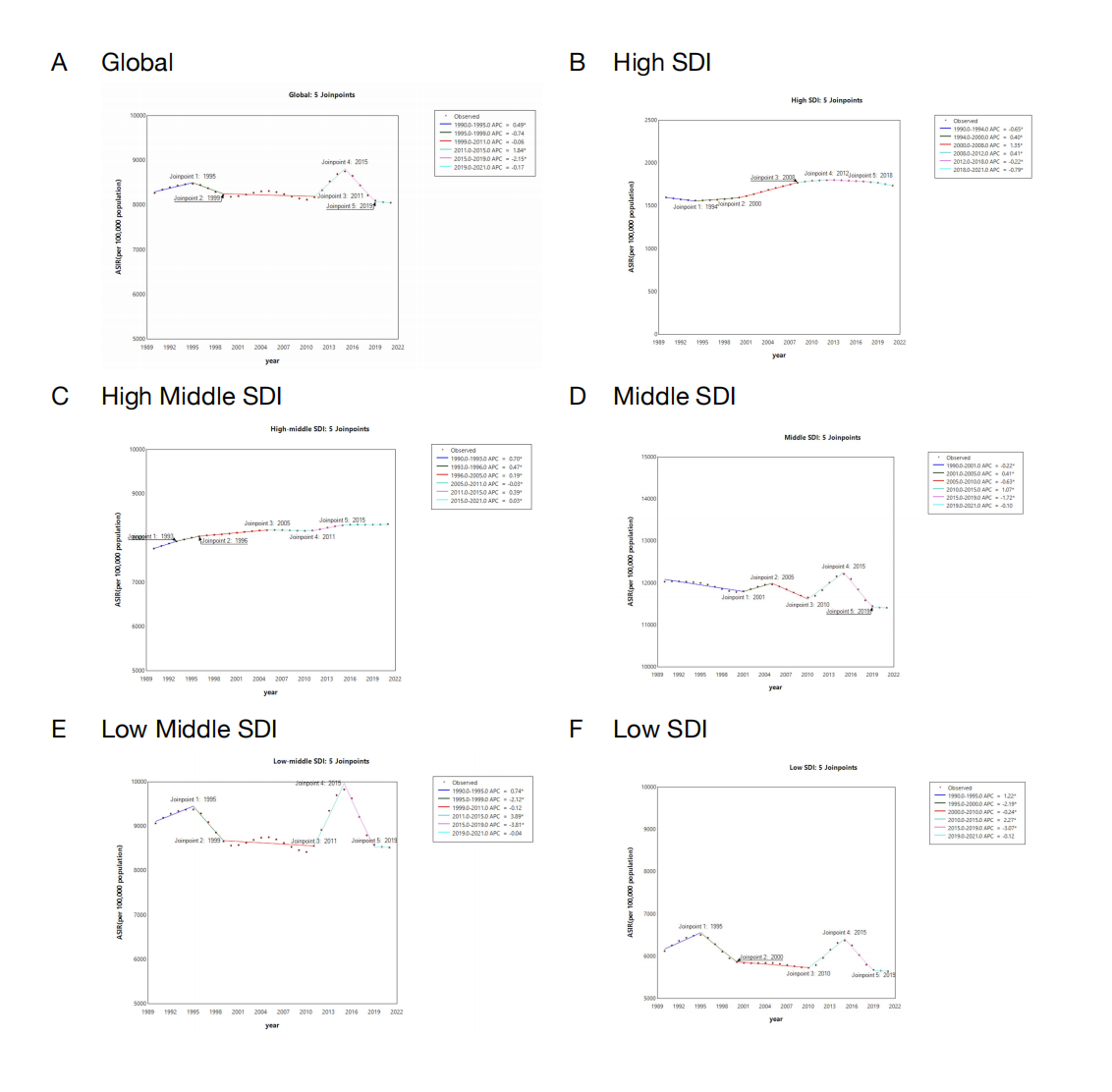

Supplement: S1 Fig — (TIF) [file pntd.0014237.s001.tif]
