# Supplementary material for: Time trends in the burden of scabies from 1990 to 2021, and projections to 2050: Insights based on the Global Burden of Disease Study 2021
Source: PLoS Negl Trop Dis. 2026 Jul 10;20(7):e0014237. doi: 10.1371/journal.pntd.0014237 (PMC13412055; doi:10.1371/journal.pntd.0014237)
Supplement: S1 Table — (DOCX) [file pntd.0014237.s002.docx]

Table S2. The global burden of scabies by SDI groups in 2021.

| **Group by SDI** | **Group by sex** | **Incidence** | | **Prevalence** | | **DALYs (Disability-Adjusted Life Years)** | | |
| --- | --- | --- | --- | --- | --- | --- | --- | --- |
|  |  | **No.(95%UI)** | **Age-specific rate per 100000(95%)** | **No.(95%UI)** | **Age-specific rate per 100000(95%)** | | **No.(95%UI)** | **Age-specific rate per 100000(95%)** |
| **Global** | | | | | | | | |
|  | **Male** | **318054349 (284005677,355054513)** | **8155.14 (7249.91,9139.24)** | **105521278 (93990096,118412588)** | **2701.15 (2397.04,3033.4)** | **2727105 (1520039,4494857)** | | **69.84 (38.87,115.22)** |
|  | **Female** | **304419226 (271885996,339619693)** | **7942.89 (7072.69,8918.24)** | **101028367 (90103237,113463905)** | **2631.45 (2341.62,2959.7)** | **2588414 (1451131,4245630)** | | **67.59 (37.83,111.26)** |
|  | **Both** | **622473574 (556234883,694992043)** | **8049.53 (7165.24,9024.24)** | **206549645 (184175477,231740498)** | **2666.47 (2368.24,2994.51)** | **5315519 (2968359,8740280)** | | **68.72 (38.33,113.25)** |
| **High SDI** | | | | | | | | |
|  | **Male** | **9265374 (8313336,10264929)** | **1796.05 (1603.63,2007.6)** | **3079629 (2763258,3431878)** | **595.3 (530.43,670.26)** | **79134 (44823,128169)** | | **15.42 (8.72,25.32)** |
|  | **Female** | **8611869 (7733728,9580520)** | **1675.29 (1500.51,1867.54)** | **2858962 (2562990,3172683)** | **554.8 (494.67,623.46)** | **72751 (41154,116272)** | | **14.29 (8.04,22.97)** |
|  | **Both** | **17877244 (16076500,19879409)** | **1737.04 (1552.82,1937.97)** | **5938591 (5327897,6604561)** | **575.51 (513.47,647.22)** | **151885 (86118,243655)** | | **14.87 (8.37,24.17)** |
| **High-middle SDI** | | | | | | | | |
|  | **Male** | **53276783 (47531929,59253351)** | **8669.57 (7728.46,9710.93)** | **17691970 (15793440,19722899)** | **2866.93 (2553.88,3228.99)** | **456279 (258208,741476)** | | **74.34 (41.63,122.29)** |
|  | **Female** | **47788887 (42766951,53199958)** | **7946.23 (7073.26,8887.06)** | **15865827 (14182434,17628007)** | **2627.59 (2337.22,2957.02)** | **405701 (229845,657612)** | | **67.84 (38.19,111.9)** |
|  | **Both** | **101065671 (90201546,112618992)** | **8312.81 (7413.35,9310.96)** | **33557796 (29927726,37366633)** | **2748.91 (2443.64,3093.74)** | **861980 (486463,1399734)** | | **71.14 (39.84,117.17)** |
| **Middle SDI** | | | | | | | | |
|  | **Male** | **137574494 (123171145,153918240)** | **11551.97 (10294.09,12978.46)** | **45665107 (40738283,51153972)** | **3821.95 (3406.21,4278.58)** | **1179887 (659788,1936151)** | | **98.86 (55.04,162.71)** |
|  | **Female** | **131105804 (117426245,145964117)** | **11256.7 (10047.82,12614.2)** | **43520624 (38839531,48631834)** | **3724.31 (3317.89,4169.22)** | **1114493 (628040,1817876)** | | **95.71 (53.68,157.08)** |
|  | **Both** | **268680297 (240226207,299946245)** | **11409.13 (10174.5,12796.17)** | **89185732 (79610620,99815563)** | **3774.71 (3361.32,4223.75)** | **2294380 (1287907,3754027)** | | **97.32 (54.38,159.96)** |
| **Low-middle SDI** | | | | | | | | |
|  | **Male** | **83595556 (73459799,94650606)** | **8455.14 (7479.01,9508.13)** | **27699468 (24152521,31605520)** | **2797.96 (2469.33,3153.87)** | **716845 (396402,1185526)** | | **72.13 (39.98,118.91)** |
|  | **Female** | **83182290 (73313779,94626904)** | **8586.21 (7580.3,9659.32)** | **27583112 (24094364,31428791)** | **2842.42 (2507.08,3203.43)** | **707348 (394406,1169292)** | | **72.66 (40.53,119.46)** |
|  | **Both** | **166777845 (146956624,189229173)** | **8521.66 (7528.21,9581.87)** | **55282581 (48210375,63002854)** | **2820.52 (2488.56,3176.97)** | **1424193 (790972,2354784)** | | **72.4 (40.25,119.19)** |
| **Low SDI** | | | | | | | | |
|  | **Male** | **34050028 (29555555,38988132)** | **5690.21 (5081.57,6404.59)** | **11288114 (9800044,12977764)** | **1892.85 (1677.05,2135.01)** | **292453（161529，484319）** | | **48.65 (27.09,79.81)** |
|  | **Female** | **33451032 (29162890,38493407)** | **5607.91 (4997.3,6293.88)** | **11107078 (9628163,12773352)** | **1866.98 (1650.81,2106.19)** | **285742（158040，473918）** | | **47.59 (26.52,78.05)** |
|  | **Both** | **67501060 (58688542,77417424)** | **5648.39 (5034.53,6348.26)** | **22395192 (19434928,25751101)** | **1879.69 (1662.82,2122.73)** | **578195（319569，959506）** | | **48.11 (26.8,78.98)** |
| **Abbreviations:DALYs,Disability-Adjusted Life Years;UI,uncertainty interval;SDI,social demographic index** | | | | | | | | |
